# Bio-Based Nano-Enabled Cosmetic Formulations for the Treatment of *Cutibacterium acnes*-Associated Skin Infections

**DOI:** 10.3390/antiox12020432

**Published:** 2023-02-09

**Authors:** Kristina Ivanova, Eva Ramon, Aleksandra Ivanova, Susana Sanchez-Gomez, Tzanko Tzanov

**Affiliations:** 1 Grup de Biotecnologia Molecular i Industrial, Department of Chemical Engineering, Universitat Politècnica de Catalunya, Rambla Sant Nebridi 22, 08222 Terrassa, Spain; 2 Bionanoplus S.L., Pol. Mocholi, Plaza Cein 5, Nave B14, 31110 Noain, Spain

**Keywords:** oregano oil, zein nanocapsules, pH-controlled cargo release, cosmetic formulations, antibacterial and antioxidant nanocapsules, *Acne vulgaris* treatment

## Abstract

Acne is a common chronic skin condition with serious physical and psychosocial consequences. In some cases, the appearance of pimples, whiteheads, or blackheads on the face, neck, and back may lead to scarring, disfiguring, depression, frustration, and anxiety in patients. Current treatments rely on antibiotics to eradicate *Cutibacterium acnes* (*C. acnes*), the bacterium responsible for this skin condition. However, these approaches do not scavenge the reactive oxidative species (ROS) generated during disease development and raise concerns about the increase in antimicrobial resistance. In this study, an environmentally friendly and cost-effective self-assembly nanoencapsulation technology based on zein, a bio-based hydrophobic protein, was employed to produce multifunctional essential oil (EO)-loaded nanocapsules (NCs) with superior antioxidant and bactericidal activity toward *C. acnes*. The NCs displayed “smart” release of the active cargo only under the conditions that were conducive to acne proliferation on skin. Once incorporated into creams, the EO-loaded NCs led to a complete inhibition of *C. acnes* and demonstrated the capacity to scavenge ROS, thus preventing damage to human skin cells. The in vitro permeation studies revealed that the nanoformulated EO was able to penetrate through the epidermis, indicating its potential for the treatment of skin diseases, such as acne.

## 1. Introduction

*Acne vulgaris*, or acne, is a chronic skin disorder affecting 95% of the global population, and it is considered the eighth prevalent health condition worldwide [1,2]. This disease starts as soon as age nine and continues into adulthood, and it has huge psychosocial impact on individuals. Affected patients exhibit comedones, nodules, pustules, and scarring on the face and the upper part of the body, frequently associated with psychological issues, such as dissatisfaction with one’s appearance, lack of self-confidence, lower self-estimation, and social dysfunction. The psychological sequelae of acne also include increased risk of insomnia, anxiety, depression, suicidal thoughts, and even suicide [3,4].

Acne is a multifactorial disorder caused by sebum over-production in skin cells, hormonal changes, and microbiome imbalance [5,6]. This disease is triggered by an overlapping of several events in the pilosebaceous follicles, such as hyperproliferation of the Gram-positive anaerobic bacterium *Cutibacterium acnes* (*C. acnes*) and the presence of reactive oxidative species (ROS), e.g., nitrous oxide, hydroxyl, and superoxide, that are generated as the side products of the host immune response and sebum oxidation by *C. acnes* [7].

Current topical treatments, which are based on comedolytic agents (e.g., retinoids, benzoyl peroxide, and salicylic acid) or antibiotics (e.g., clindamycin and erythromycin) for controlling the colonization of *C. acnes*, frequently aggravate skin irritation and inflammation. These approaches are not effective because they (i) do not present antioxidant properties that are able to reduce ROS harmful effects; (ii) do not pass through the skin epidermis; (iii) cause side effects, such as toxicity and skin drying; and (iv) over long periods, with the use of antibiotics in particular, can lead to the appearance of resistance strains, jeopardizing not only *acne vulgaris* treatment outcomes, but also other *C. acnes*-associated diseases, e.g., infective endocarditis [8,9]. Natural extracts from plants’ leaves, flowers, roots, or stems have been introduced as alternative and more effective solutions for managing *acne vulgaris*. Aloe, tea tree oil, propolis, rosemary, and chamomile are largely employed as they feature strong antioxidant properties and bactericidal activity towards *C. acnes,* thus implying less selective pressure for antimicrobial resistance (AMR) appearance [10,11,12]. Despite their prominent anti-*C. acnes* potential, these bio-actives show uncontrolled biodegradability and induce toxicity and inflammation.

Advances in nanotechnology have allowed the redesigning of natural actives, potentiating their activity and stability [13]. Nanocarriers, including liposomes, niosomes, solid lipid nanoparticles, and micro-emulsions, provide protection for the encapsulated hydrophilic and lipophilic actives, target and control delivery while minimizing their toxicity toward other tissues, and allow higher skin penetration and time residence. Nanoformulation changes the chemical and physical properties of natural actives, endowing them with superior antibacterial performance and long-term stability when compared to their free form. The surface area-to-volume ratio increases the interaction with bacterial cells, causing cellular damage at lower dosages in contrast to non-encapsulated actives [14,15,16].

In this study, we developed novel anti-acne formulations comprising multifunctional zein-based nanocapsules (NCs) loaded with plant-derived antibacterial and antioxidant essential oil (EO) for efficient *acne vulgaris* treatment, targeting simultaneously the inhibition of ROS production and *C. acnes* overgrowth. Stable oregano oil NCs (EO-loaded NCs) were generated using a proprietary self-assembling encapsulation technology based on zein, a hydrophobic protein found in corn seed, which is included in the US FDA (Food and Drug Administration) Inactive Ingredients Database and classified as GRAS (Generally Recognized as Safe). This environmentally friendly, scalable, and cost-effective self-assembling encapsulation method yielded EO-loaded nano-delivery vehicles without the need for additional downstream processes, such as purification or drying steps. The use of a highly lipophilic and biodegradable zein protein is anticipated to enhance stability and impart controlled release of poorly water-soluble EO actives [17]. Nano-transformation will additionally boost the anti-acne performance of EO due to improved interaction and disruption of bacterial cells at a lower non-toxic concentration [14,18]. Incorporating EO-loaded NCs into cosmetic formulation will outperform conventional anti-acne products in terms of skin permeation and enhanced antibacterial efficacy. The innovative nano-cosmeceuticals were validated for their potential to inhibit *C. acnes* and to scavenge free radicals in vitro. The biocompatibility and antioxidant activity of the nano-enabled formulations were assessed with human skin cells. The release profile and transdermal delivery of the NCs upon a specific trigger of acne infection, such as pH 7, were evaluated using Franz cells.

## 2. Materials and Methods

### 2.1. Materials

Zein, a protein extracted from maize, was purchased from Flo Chemical Corporation (Ashburnham, MA, USA) and employed for NC generation. Oregano oil from Thymbra capitata (100% pure) was kindly provided by the TELIC S.A. (Barcelona, Spain). *Cutibacterium acnes* CECT 5684 was obtained from the Spanish Type Culture Collection (CECT, Valencia, Spain). A reinforced clostridial medium, obtained from Sigma-Aldrich (Madrid, Spain), was used for *C. acnes* cultivation in all antimicrobial tests. Brain Heart Infusion (BHI) agar for bacterial culturing and viable cell counting was also purchased from Sigma-Aldrich (Madrid, Spain). Dulbecco’s Modified Eagle Medium (DMEM) media; Hank’s Balanced Salt Solution (HBBS); Fetal Serum Bovine (FBS); mixture of 5000 Units penicillin (Pen), 5 mg mL^−1^ streptomycin (Strp), 200 mM of glutamin (Gln), and 10 mM of phosphate buffer at a pH of 7 (PBS) were provided by Sigma-Aldrich (Madrid, Spain). Human keratinocytes cells (HaCaT) were purchased from the American Type Culture Collection (Manassas, VA, USA). The AlamarBlue Cell Viability Reagent and GasPak^TM^ EZ to induce a microaerophilic/CO_2_-enriched environment were supplied by Thermo Fisher Scientific (Barcelona, Spain). HCl, oleic acid, and Tween 20 were provided by Panreac Applichem (Barcelona, Spain). All other reagents were purchased from Sigma-Aldrich if not specified otherwise.

### 2.2. Preparation of EO NCs

NCs containing oregano oil were prepared as described previously [19]. Briefly, 0.2% (*w/w*) zein and 9.8% (*w/w*) propylene glycol were mixed until complete dissolution and further incorporated into a homogeneous mixture of 6% (*w/w*) propanediol (DuPont Tate & Lyle Bioproducts, Loudon. TN, USA), 6% (*w/w*) denatured alcohol (Barcelona, Spain), 6% (*w/w*) Tween 20, 0.8% (*w/w*) oleic acid, 0.01% (*w/w*) HCl, and 0.5% (*w/w*) oregano oil. The resultant surfactant–EO–zein mixture (29 g) was added dropwise under continuous agitation into water (71 g) to generate the NCs [20].

### 2.3. Characterization of EO-Loaded NCs

The NCs were characterized in terms of their macroscopic appearance, e.g., absence of aggregates and precipitates. The NCs’ surface charge, size, and polydispersity were determined using a Zetasizer Nano ZS (Malvern Instruments Inc., Malvern, Worcestershire, UK). The number of NCs per milliliter were assessed by Nanoparticles Tracking Analysis using the NanoSight NS 300 (Malvern Instruments Inc., UK) in the flow mode and the software NTA 3.2 to capture several frames of the NC suspension. The size and the morphology of the NCs were also studied using a Transmission Electron Microscopy, TEM (Tecnai G2 F20, FEI company, Hillsboro, OR, USA), at 80 kV acceleration voltage. Prior to observation, 10 µL of the samples were placed on ultrathin carbon on holey carbon grids and air dried.

The amount of EO encapsulated in the NCs was assessed by carvacrol quantification using high performance liquid chromatography (HPLC). The HPLC experiments were performed using an Agilent 1260 Infinity system equipped with a quaternary pump, an auto-sampler with a high-sensitivity cell, a thermostatted column compartment, and a diode array detector. The ultraviolet (UV) spectra were collected at 220 and 278 nm. The Agilent OpenLab CDS software version 2.2 was used for instrument control, data collection, and data processing. The column used was a Zorbax SB-C18 (250 × 4.6 mm, 5 µm). The mobile phase was an isocratic combination of acetonitrile:water (50:50) with a flow rate of 2 mL min^−1^. The injection volume for all samples and standard solutions was 10 µL. The encapsulation efficiency (EE) was calculated as follows:

EE (%) = (C_total_ − C_non-encapsulated_)/C_total_ × 100, (1)

where C_total_ is the total carvacrol concentration in the suspension, and C_non-encapsulated_ is the concentration of the “non-encapsulated” active—the filtrate collected after NC centrifugation for 30 min at 4000× *g* using Amicon^®^ centrifugal filters (100 kDa cut-off, Millipore, Madrid, Spain).

The EO release from the NCs was assessed in PBS at a pH of 5.5 and at a pH of 7 for 4 h at 32 °C (the temperature of the skin). A total of 0.2 g of the NCs was redispersed in 10 mL of PBS, at a pH of 5.5 or 7, and incubated at 32 °C with shaking (30 rpm). At defined time intervals, 500 µL of each sample was collected and replaced with fresh PBS. The collected samples were centrifugated for 30 min at 4000× *g* using Amicon^®^ centrifugal filters (100 kDa cut-off, Millipore, Madrid, Spain), and the filtrates were subjected to HPLC analysis to quantify the amount of EO released.

### 2.4. Antibacterial Activity of EO-Loaded NCs

*Broth Microdilution Test.* The minimal inhibitory concentration (MIC) of the NCs and the bulk EO was determined by the standardized broth microdilution method. *C. acnes* was grown in the reinforced clostridial medium under anaerobic conditions using an anaerobic container system at 37 °C for 3 days. For the antibacterial tests, the turbidity of the bacterial suspension was adjusted to an optical density of 0.01 at 600 nm in the reinforced clostridial medium, corresponding to 1 × 10^6^ colony-forming units (CFU mL^−1^). A total of 50 µL of the *C. acnes* suspension was mixed with 50 µL of the EO-loaded NCs and the bulk EO at different concentrations (0.0125–0.2 EO % (*v/v*)) in a sterile 96-round-bottom-well plate and incubated in an anaerobic environment at 37 °C for 24 h. The reinforced clostridial medium served as a negative control. The lowest concentration required to inhibit *C. acnes* growth was defined as the MIC. Each sample was tested in a triplicate.

*Time-kill kinetic assay.* The minimal time necessary for the NCs to completely eradicate *C. acnes* was determined using a time-kill kinetic assay. The *C. acnes* suspension was diluted in a sterile phosphate-buffered saline at a pH of 7 (PBS) to an OD = 0.01 at λ = 600 nm (~10^6^ CFU mL^−1^). For the assay, 250 μL of the bacterial inoculum was mixed with 250 μL of the samples and incubated at 37 °C under anaerobic conditions. At different time points, 15 μL of the samples was taken, and the survived bacteria were enumerated on a BHI agar using the drop plate method. *C. acnes* in the PBS was used as a negative control (no bactericidal activity).

### 2.5. Formulation of Anti-Acne Cosmetics Containing EO-Loaded NCs

Anti-acne cosmetic formulation was prepared by incorporating the encapsulated EO (0.02%) into a cosmetic facial cream base containing water, glycerin, and dimethicone. The antimicrobial activity of the nano-enabled anti-acne cream was assessed and compared to the cream with the bulk EO. The creams were prepared using a standard reverse emulsification method. An aqueous phase containing 5 g of triethanolamine was added dropwise into an oil phase containing 30 g of stearic acid, 5 g of liquid paraffin, 3 g of white beeswax, and 2 g of the NCs, while the mixture was stirred at 400 rpm. The temperature of the aqueous phase and the oil phase was adjusted to 70 and 65 °C, respectively. The mixture was stirred at 200 rpm (C-MAG HS7, IKA^®^, Staufen, Germany) and allowed to cool down to room temperature to form the creams.

### 2.6. Antibacterial Activity of the Nano-Enabled Cosmetic Formulations

The antibacterial activity of the creams was evaluated by using an agar diffusion test. *C. acnes* was grown in the reinforced clostridial broth for 3 days under anaerobic conditions at 37 °C. Afterwards, a BHI agar was inoculated with *C. acnes* (~10^8^ CFU mL^−1^), and 25 mL was poured into sterile plastic petri dishes. The agar was allowed to solidify, and 3 holes with a diameter of 7 mm were punched aseptically with a sterile cork borer. Then, 0.1 g of the samples were placed into the wells, and the plates were incubated at 37 °C under anaerobic conditions for 24–48 h. Then, the antimicrobial activity of the creams was assessed by measuring the diameter of the zones of inhibition that appeared as clear zones. All diffusion tests were performed in two independent experiments, and antibacterial activity was expressed as mean ± standard deviation.

### 2.7. Radical Scavenging Activity of the Anti-Acne Creams

The antioxidant activity of the NC-containing cream was determined based on their scavenging activity over ROS using 1 diphenyl-2-picryl hydrazyl (DPPH) free radical in a methanol solution. A total of 0.1 mL of the sample solution was added to 3.9 mL of 60 µM of the DPPH free radical solution. After 1 h in the dark, the absorbance of the preparations was measured at 517 nm (Abs_517_) and compared to the corresponding absorbance of the blank (DPPH without the sample). Ascorbic acid at 0.5 mg mL^−1^ was used as a positive control. The % of inhibition was calculated as follows:

Radical scavenging activity (%) = ((Abs_517 blank_ − Abs_517 scavenging activity sample_)/(Abs_517 blank_)) × 100 (2)

### 2.8. Biocompatibility of the Anti-Acne Creams

The cells were seeded at a density of 6 × 10^4^ cells per well on a 96-well tissue culture-treated polystyrene plate. On the next day, the cells were exposed to 3% creams diluted in a DMEM media containing 10% FBS, 2 mM of Gln, 50 Units of Pen, and 50 mg of Strp per mL at a pH of 7.5 (complete DMEM media) and incubated at 37 °C in a humidified atmosphere with 5% CO_2_ for 2 h. After the indicated time of incubation, the NCs were removed, and the cells were washed with 200 µL of PBS and covered with 100 µL of 10% (*v/v*) AlamarBlue^®^ reagent diluted in the complete DMEM media. After 4 h at 37 °C, the absorbance at 570 nm was measured, using 600 nm as a reference wavelength, in a TECAN infinite M200 (Tecan Group, Switzerland). Resazurin, the active ingredient of AlamarBlue^®^ reagent, is non-toxic, cell-permeable compound that is blue in color, and when it is reduced to resorufin by viable cells, it becomes red. The quantity of the resorufin formed is directly proportional to the number of viable cells. The relative viability (%) of HaCaT cells was determined for each concentration of the creams with EO-loaded NCs and compared with the cells incubated with only a cell culture medium. All tests were performed in triplicate. Additionally, the cells were also subjected to a cytotoxicity assessment using the Live/Dead Viability/Cytotoxicity Kit for mammalian cells (Molecular Probes, ThermoFisher Scientific, Madrid, Spain). This kit includes two fluorescent probes, calcein and ethidium homodimer-1, that provide simultaneous determination of live (green fluorescence) and dead cells (red fluorescence) [19]. The cells were stained with a mixture of both stains in a ratio of calcein:ethidium homodimer of (4:1) in PBS for 15 min in the dark and were visualized using a fluorescence microscopy at 480/500 nm for calcein and at 490/635 nm for ethidium homodimer.

### 2.9. Intracellular ROS Measurement

This method uses HaCaT cells and allows for a more realistic screening of antioxidant activity against ROS than other methods using stable free-radical molecules, such as ABTS or DPPH [21]. Briefly, the cells were seeded at a density of 6 × 10^4^ cells in 100 μL of complete DMEM media per well on a black, clear-bottom, 96-well microplate. Next day, the growth medium was removed, and the cells were washed with 200 μL of PBS. The cells were treated per triplicate for 2 h with 100 μL of 3% of cream formulations diluted in HBSS at a pH of 7 and 25 μM of 2′,7′-dichlorofluorescein diacetate. Ascorbic acid (150 μg mL^−1^) was used as a positive control for antioxidant activity. The cells were further washed with 200 μL of PBS, and, subsequently, oxidative stress was induced by the addition of 600 μM of 2,2′-azobis(2-methylpropionamidine) dihydrochloride in 100 μL of HBSS. Immediately, the 96-well microplate was placed into a TECAN infinite M200 (Tecan Group, Männedorf, Switzerland). Emission at 535 nm was measured after excitation at 485 nm for 1 h every 5 min. After the subtraction of the initial fluorescence values, the area under the curve for fluorescence versus time was integrated to calculate the CAA value as follows:

(3) $$CAA\;(\%)=1-\left(\frac{\int SA}{\int CA}\right)$$

where ∫SA is the integrated area under the curve of the sample fluorescence versus time, and ∫CA is the integrated area from the control curve. The CAA values (%) are presented as mean ± SD for the triplicate data from one experiment.

### 2.10. EO Permeation Studies

Dermatomized porcine skin (400 μm thickness) was used for the permeation studies using Franz diffusion cells. At first, the integrity of the skin was confirmed by its visual inspection and transepidermal water loss measurements (Tewameter^®^ TM 300). Then, the dermatomized skin was mounted on the Franz diffusion cells with a 1.767 cm^2^ cross-sectional area and 7 mL receptor volume. The anti-acne formulations (*n* = 5) were applied onto the skin in the donor compartment, and the system was maintained at 32 ± 1 °C with the help of a thermo-regulated outer water jacket, while the diffusion medium (PBS, pH 7) was stirred continuously using a magnetic stirrer. Finally, the system was disassembled, and the concentration of EO in the skin was quantified by HPLC.

### 2.11. Statistical Analysis

All data are presented as mean ± standard deviation. For multiple comparisons, statistical analysis was performed using a one-way analysis of variance (ANOVA), with *p* values less than 0.05 considered as statistically significant.

## 3. Results and Discussion

In this work, EO was nanoformulated using a proprietary self-assembling technology based on zein and non-volatile solvent propylene glycol [20]. Zein is a water-insoluble maize protein with amphiphilic characteristics, which is able to self-assemble into nano- and micro-structures. This protein has been used as a platform for encapsulating a range of hydrophobic and hydrophilic actives, including heparin, curcumin, α-tocopherol, and EO, achieving high loading capacity coupled with enhanced stability, sustained release, photoprotection, and bioavailability [22,23,24,25].

### 3.1. NP Characterization

**Figure 1:**
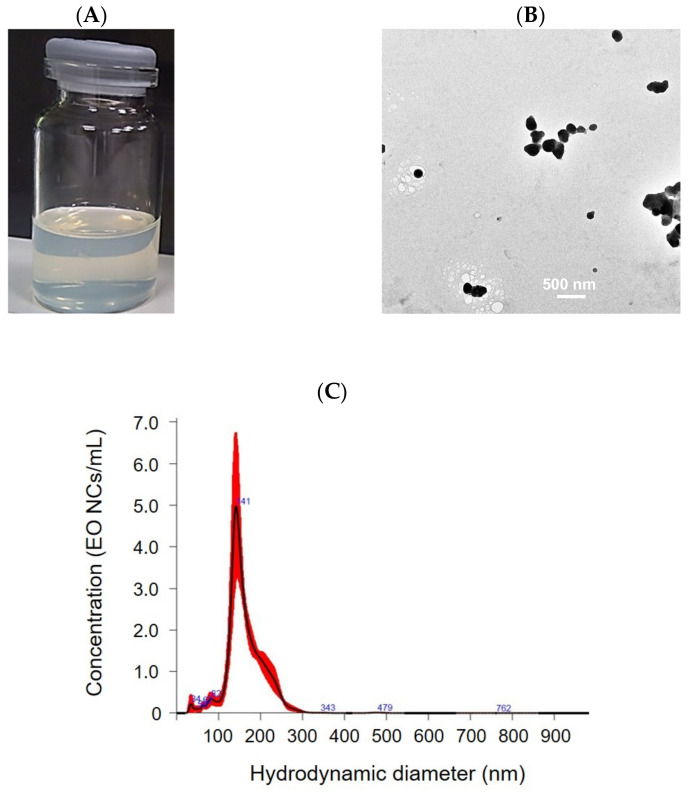
Characterization of EO-loaded NCs: (**A**) macroscopic appearance, (**B**) TEM images, and (**C**) size distribution.

The obtained EO-loaded NC suspension appears opalescent-transparent and yellowish, due to the zein, containing a high concentration of the yellow pigment xanthophyll (Figure 1A) [26]. The NCs are spherical in shape with an average size of 164 ± 5 nm and a narrow particle size distribution (0.4 ± 0.004). The NCs’ concentration, as determined by NTA, is 3 × 10^9^ NCs mL^−1^. The EO-loaded NCs possess relatively high positive ζ-potential of 21 ± 2 mV, indicating good colloidal stability. Visible aggregate formation and phase separation due to NC precipitation were not observed even after 12 months of storage at room temperature. The size and ζ-potential values of the NCs did not change, further confirming the long-term storage stability of the NCs (Figure 1). This is explained by the use of the non-ionic surfactant Tween 20 in the NC formulation, enhancing the electrostatic and steric repulsion against aggregation. Since carvacrol represents 73.8% of the EO composition, this was used as a standard to determine the EE [27]. The HPLC analysis determined a 86 ± 1% EO encapsulation yield.

### 3.2. In Vitro EO Release

**Figure 2:**
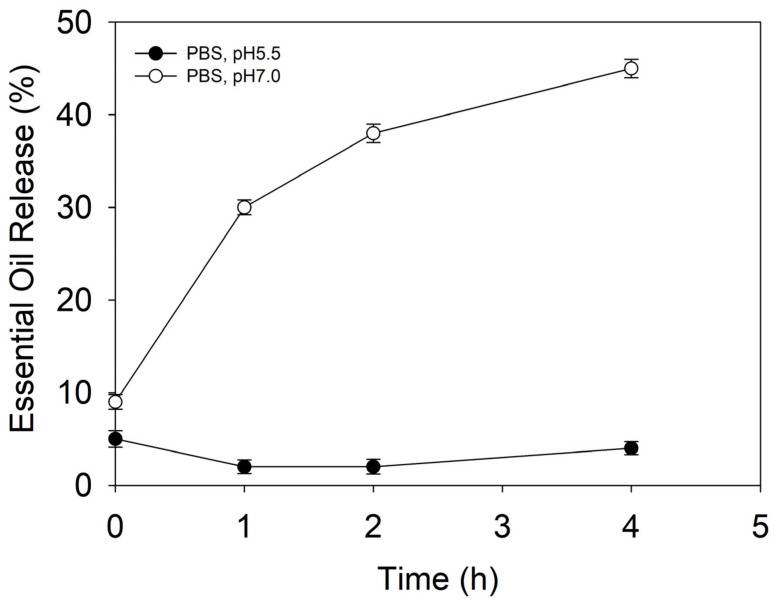
EO release from the NCs under conditions mimicking healthy skin (pH 5.5) and conditioned skin (pH 7) promoting *acne vulgaris* occurrence.

Maintaining a pH between 5–6 is vital for normal skin functioning and homeostasis. Higher pHs cause changes in healthy skin’s microbiome balance and promote *C. acnes* proliferation and occurrence of *acne vulgaris* [28]. The release behavior of the NCs was assessed at a pH of 5.5 and a pH of 7, mimicking the physiological conditions in healthy and conditioned skin, respectively. At defined time intervals, the percentage of EO released from the NCs was quantified by HPLC (Figure 2). At a pH of 5.5, the NCs did not show any EO leakage, indicating good stability and retention of actives. Increasing the pH to 7, however, triggered the release, and approximately 45% of the encapsulated EO was liberated from the NCs within 4 h of incubation, which was attributed to pH-induced destabilization and rearrangement of the zein’s secondary structure [28,29,30].

### 3.3. Antimicrobial Activity of EO-Loaded NCs against C. acnes

**Figure 3:**
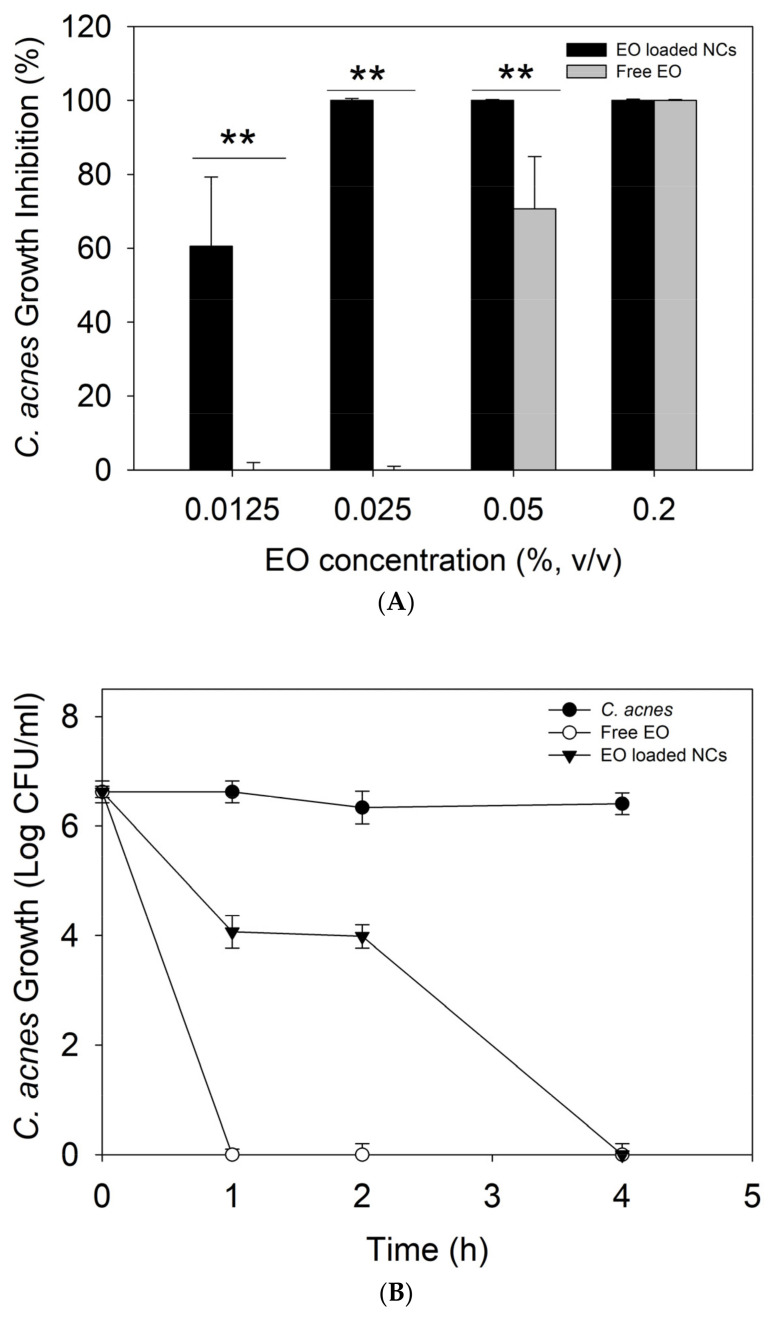

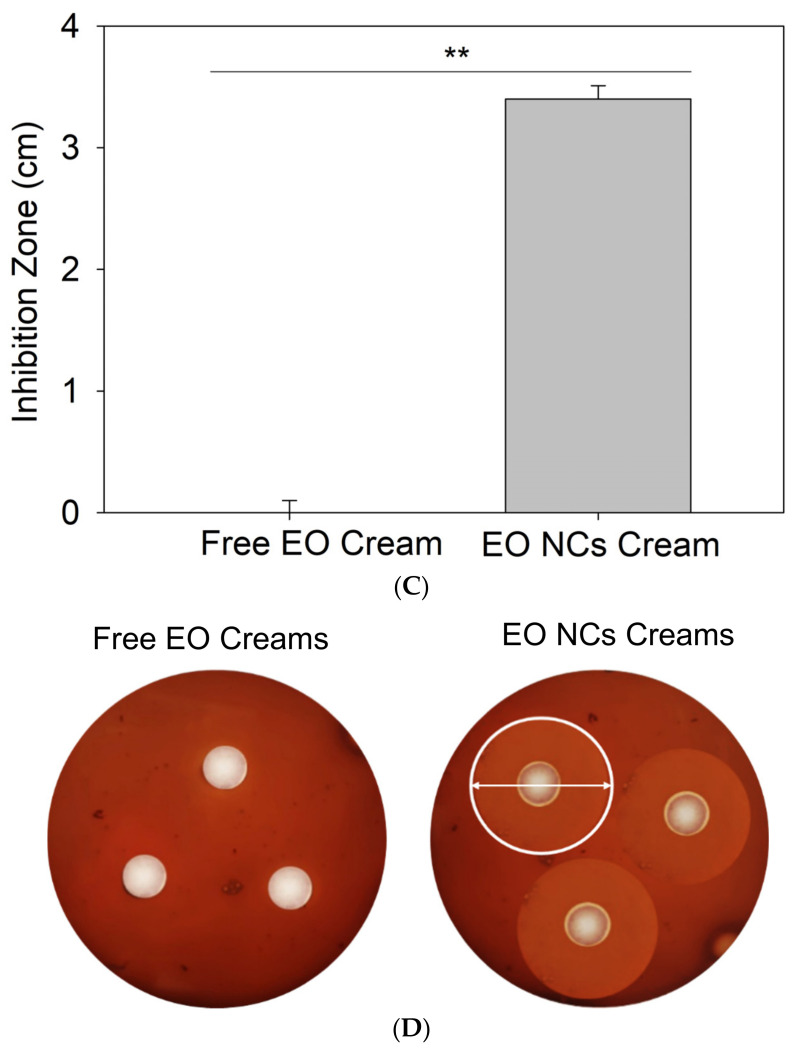
(**A**) Antibacterial activity of non-encapsulated and encapsulated EO against *C. acnes.* (**B**) Time-kill curves, (**C**) zone of inhibition (cm) of *C. acnes* growth by the free EO or EO-loaded NC anti-acne creams, and (**D**) agar plates inoculated with *C. acnes* and the free EO or EO-loaded NC anti-acne creams. All experiments were performed in triplicate, and the data are expressed as the mean ± standard deviation. ** stands for *p* < 0.05, considered statistically significant.

Plant-derived antimicrobials have gained significant attention as being more efficient against both susceptible and resistant bacteria than other antibiotic alternatives, with lower potential for resistance development. Although the strong anti-*C. acnes* activity of natural oils, such as cloves, cactus, and tea tree, has been previously described, their enhanced bactericidal effect due to the nano-size transformation has not yet been reported [31]. Our results demonstrated up to 8-fold increase in the antimicrobial activity of oregano oil against *C. acnes* upon nanoformulation (*p* < 0.05). A complete inhibition (100%) of bacterial growth was observed for the EO-loaded NCs at 0.025% (*v/v*) of the oil, while 0.2% of the bulk active was needed to achieve the same effect (Figure 3A). It is noteworthy that the antibacterial effect of the empty NCs, without the EO, was also evaluated, but any activity against *C. acnes* was not observed. These results are in agreement with our previous works validating nano-scale transformation as an innovative approach potentiating the antibacterial performance of different actives, including biopolymers, EO, enzymes, and metals, mainly due to improved interaction and disturbance of bacterial membrane [19,32,33].

A time-kill kinetic assay was further performed at the MIC of the free EO and EO-loaded NCs to assess the kinetics of their bactericidal activity toward the target pathogen overtime. At the MIC, the bulk EO completely eradicated *C. acnes* within the first hour of incubation, while the EO-loaded NCs demonstrated a slower killing rate. A decrease in the live cell count by 2.6 log_10_ and 2.3 log_10_ (CFU·mL^−1^) was achieved within the first and second hours, respectively, while complete bacterial removal was reached at the fourth hour of incubation (Figure 3B). These results confirm the bactericidal effect of the developed NCs and are in agreement with the sustained release of antibacterial EO from the zein matrix as a function of specific skin condition, e.g., *C. acnes* infection (Figure 2).

The anti-*C. acnes* EO-loaded NCs were further incorporated into a commercial cream’s base formulation, and its bactericidal performance was assessed in vitro using a zone of inhibition assay. The EO-loaded NC-containing creams showed superior antibacterial efficiency against the target bacterium compared to the control, i.e., anti-acne formulation with the free active (*p* < 0.05). No zones of inhibition were observed for the cream with the bulk EO, while the nano-enabled anti-acne formulation reached an inhibition zone of 3 cm (Figure 3C).

Carvacrol, a phenolic compound present at high percentage in oil, is considered the principal active responsible for the strong antibacterial activity against *C. acnes.* The intrinsic hydrophobicity of this compound is thought to facilitate its interaction with bacterial cell through the direct binding with proteins, causing changes in the cells’ morphology and disintegration of the membrane, which lead to cellular dysfunction and death. Inactivation of essential enzymes implicated in the synthesis of structural cell components is also likely to occur. Furthermore, the improved membrane-disturbing capacity due to the nanoform potentiates the antibacterial effect of this active at lower concentrations and results in higher anti-acne performance in regard to the cream loaded with the bulk active [34].

### 3.4. Antioxidant Activity Using DPPH and Cellular Antioxidant Activity

**Figure 4:**
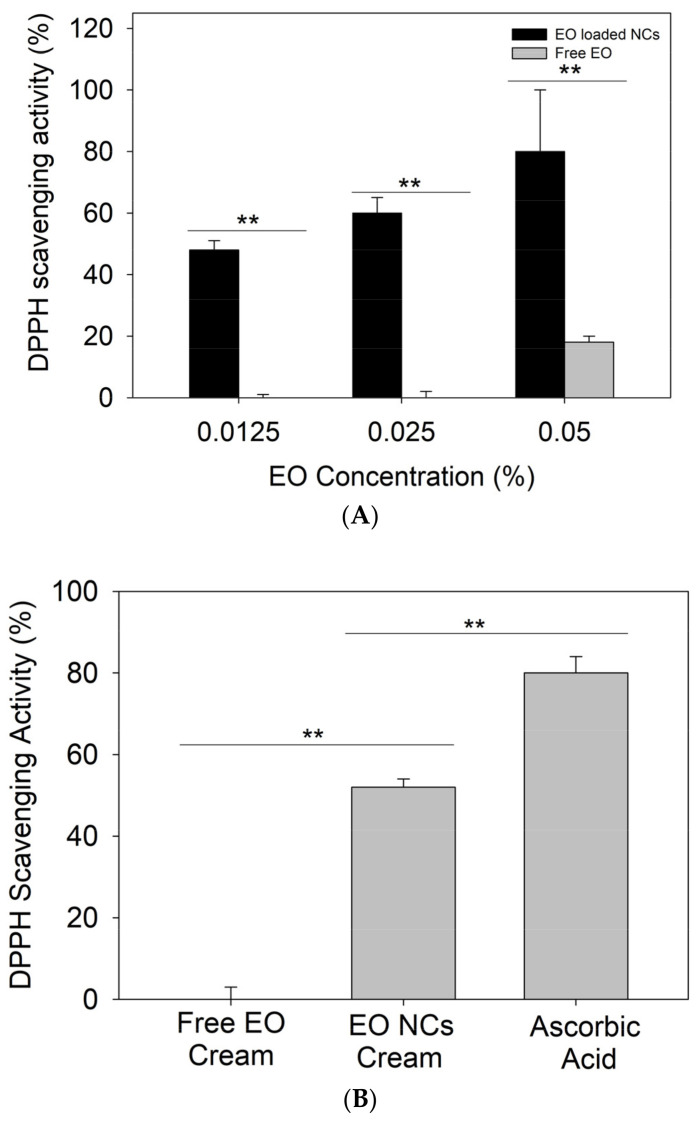

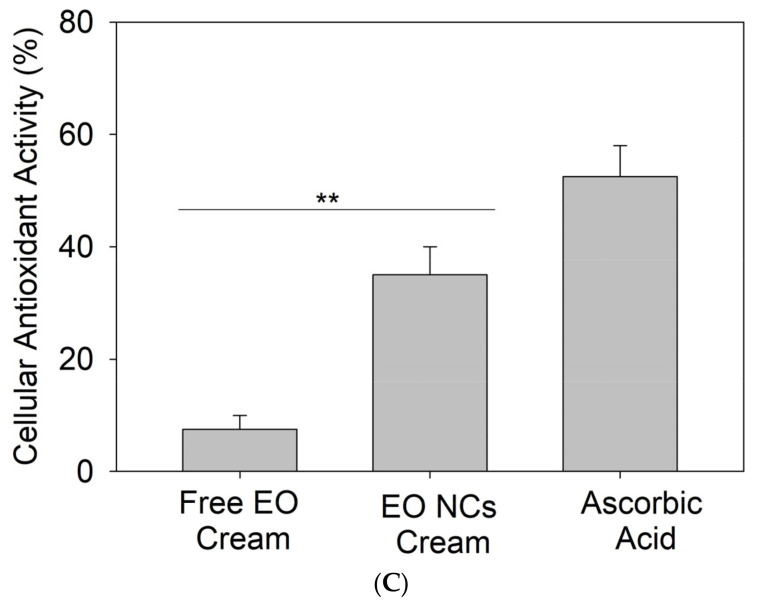
(**A**) Antioxidant activity of bulk EO and EO-loaded NCs, (**B**) free DPPH radical scavenging by the free EO and EO-loaded NC creams, and (**C**) cellular antioxidant activity of the free EO and EO-loaded NC creams. All experiments were performed in triplicate, and the data are expressed as the mean ± standard deviation. ** stands for *p* < 0.05, considered statistically significant.

Recently, several studies have demonstrated the importance of antioxidant actives in the treatment of *acne vulgaris.* Apart from the oxidative species produced by *C. acnes* creating a favorable microenvironment for bacteria growth, neutrophils’ defensive mechanisms for controlling the infection also generate reactive species [35]. This excessive amount of ROS overwhelms the natural antioxidant defense of the body, damaging the epithelium and the pilosebaceous area, and worsening the acne condition of affected individuals [12,36,37]. In addition to the enhanced antibacterial effect, higher antioxidant activity was observed for the nanoformulated EO when compared to the bulk material at the same amounts. Up to 60% of DPPH reduction was achieved by 0.0125% of encapsulated EO, while the same amount of the bulk oil did not demonstrate antioxidant properties (*p* < 0.05). The enhancement of the EO’s intrinsic radical scavenging potential upon its nanoformulation can be explained by the large surface area-to-mass ratio of the NCs and the improved absorption of the free DPPH radicals. The control zein NCs without EO possess negligible antioxidant activity (15%), further demonstrating the advantage of using nanoformulated actives. Previous studies that focused on the nanoformulation of different bio-actives already reported similar behavior [38,39]. To this end, we assessed the antioxidant activity of the developed EO-loaded NC creams. The hydrogen-donating ability was assessed using a method based on the reduction of a methanol solution of the free radical DPPH (purple color) to its non-radical form (yellow color) [40]. The results showed the increased ability of the EO-loaded NC creams to scavenge free DPPH (*p* < 0.05) due to the intrinsic antioxidant properties of the oil (Figure 4A,B) [41].

Additionally, the antioxidant activity of the NC creams was assessed in HaCaT cells as a more realistic way than the currently used “test tube” DPPH method [21]. An accumulation of ROS due to cellular oxidative metabolism or external factors can cause molecular damage, leading to cell apoptosis [42]. This assay determines the ability of compounds to penetrate the membrane and act intracellularly as antioxidants [21]. The cellular antioxidant activity assay demonstrated that the EO-loaded NC formulations displayed higher free radical scavenging potential than the control cream with the non-encapsulated active (*p* < 0.05), which is attributed to the potentiating effect of the nano-form (Figure 4A,C) [43]. The antioxidant capacity of oregano oil is associated with the phenolic compound carvacrol, which is able to reduce free radicals [21,44,45].

### 3.5. EO Permeation Studies

Percutaneous absorption studies using Franz diffusion cells were conducted with porcine skin to evaluate the degree of EO penetration through the skin. Porcine skin was employed due to its resemblance to human skin physiological and anatomical properties and the lack of concern about ethical issues arising from its use [46]. In epidermis, the outermost layer is structured in a similar keratinizing stratified organization, and the thickness ratio between the dermis and the epidermis is identical between human and pork. This ratio is important for maintaining the viscoelastic properties of the skin, which are responsible for (i) protecting the body against external microbial infection, (ii) wound healing, and (iii) recovering skin shape after a force has been applied [47].

**Figure 5:**
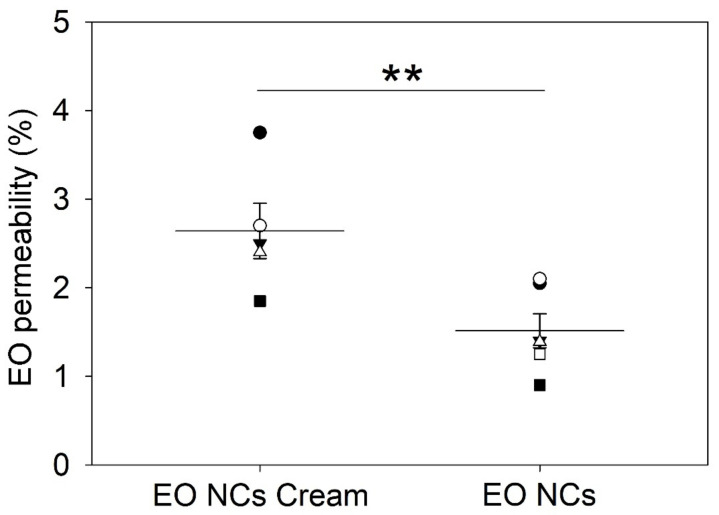
Percentage of EO permeation through the skin after 2 h of administering the EO-loaded NCs and the EO-loaded NC creams. Data are expressed as the mean of five replicates ± standard deviation. ** stands for *p* < 0.05, considered statistically significant.

The dermatomized porcine skin was placed in the Franz diffusion cells, and the amount of active released from the EO-loaded NC creams and from the EO-loaded NCs into the skin were determined. The results revealed that about 1.5 ± 0.2% and 2.6 ± 0.3% of EO of the total applied dose of NCs and NC creams, respectively, were delivered into the skin (Figure 5). The higher EO penetration for the NC-containing cosmetics than the EO-loaded NCs alone (*p* < 0.05) is attributed to the presence of permeation enhancers, such as water, alcohols, and non-ionic surfactants in both the NCs and the cream matrix. These enhancers for transdermal delivery of antibacterial and antioxidant EO diminished the barrier properties of skin and boosted the NCs to reach deeper regions of the epidermis [48].

### 3.6. Nanosafety of Anti-Acne EO-Loaded NC-Containing Cosmetic Formulations

The inclusion of nanomaterials in cosmetic products has raised some discussion about their toxicity due to size, safe dosage administration, and material composition. The high surface/volume ratio of these materials makes them more reactive than their pristine forms [49]. The nano-size allows them to easily pass through cell membranes and potentially interact with molecules that are important for cell viability (e.g., DNA and proteins) [50]. Therefore, it is critical to assess whether the developed anti-acne creams are safe toward skin cells. An in vitro viability test using keratinocytes (HaCaT cell line) was carried out to confirm the biocompatibility of the nano-enabled cosmetic products. Keratinocytes are the major cell type of the protective thin outer layer of skin epidermis, where the creams are intended to be applied [51].

**Figure 6:**
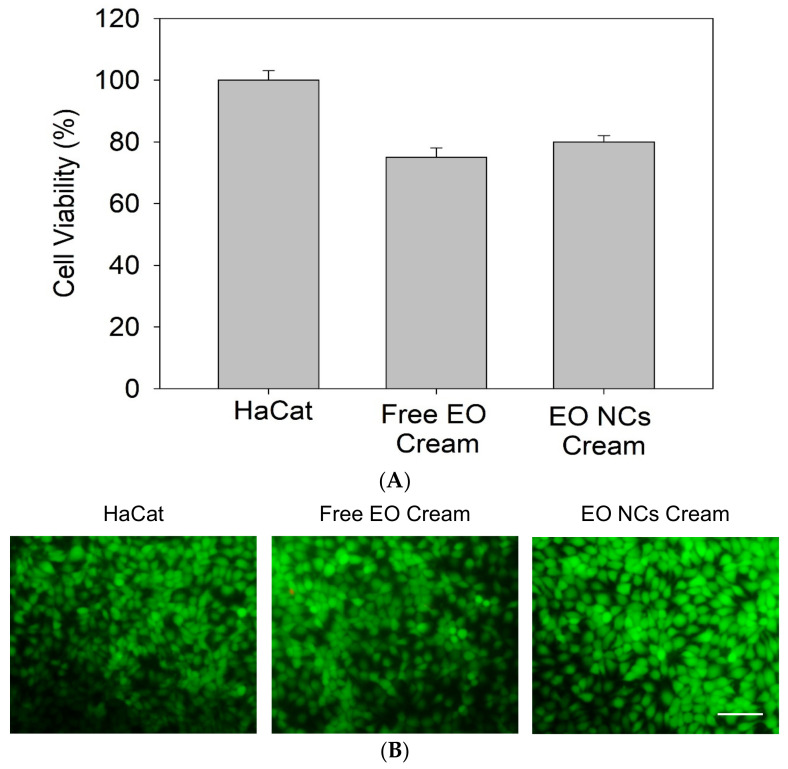
Biocompatibility of the free EO and EO-loaded NC anti-acne creams assessed by (**A**) Alamar Blue and (**B**) Live/Dead assay in HaCaT cells. All experiments were performed in triplicate, and the data are expressed as the mean ± standard deviation.

The results showed that the anti-acne formulation did not cause significant side effects on the human HaCaT cells, confirming that the EO-loaded zein-based NCs and the compounds employed for the preparation of the cosmetic base formulation are safe for use (Figure 6A). After 24 h of exposure, the cells exhibited intact membranes, allowing the intracellular retention of the green dye calcein and showing a similar behavior as the control creams with the non-encapsulated active (Figure 6B).

## 4. Conclusions

Despite the large number of marketed anti-acne products intended to reduce inflammation or *C. acnes* overgrowth, these products do not scavenge ROS produced by the host immune system’s defense mechanism against infection. In this work, we developed innovative bio-based cosmetic formulations containing EO-loaded NCs to simultaneously decrease ROS generation and eradicate *C. acnes*, which is responsible for the severity of acne disease. Taking advantage of the intrinsic EO bio-activities and zein self-assembling capability and biocompatibility, stable EO-loaded NCs with superior antioxidant and antibacterial performance than the bulk EO were synthesized. The novel multifunctional delivery nanosystems released the encapsulated actives upon a pH typical for acne skin infection and completely eradicated the pathogen within 4 h. The cosmetic cream formulations containing the bio-based anti-acne nano-actives showed higher ROS scavenging potential and enhanced bactericidal effect toward *C. acnes* than the bulk oil at concentrations that are safe to human skin cells. The compounding of EO nano-actives in cosmetic formulations increased the EO’s transdermal permeation into the skin, indicating its capability to reach inflamed areas that are colonized by *C. acnes*. The use of nanoformulated EO with improved antimicrobial capacity is an innovative approach for effective acne treatment, which is expected to impart less pressure for the selection of AMR.

## Data Availability

All data generated or analyzed during this study are included in this manuscript.
